# Association Between the Preoperative Triglyceride-Glucose Index and Intraoperative Blood Pressure Variability in Major Abdominal Surgery: A Retrospective Cohort Study

**DOI:** 10.3390/jcm15156058

**Published:** 2026-08-04

**Authors:** Hongyu Huo, Zheng Zhang, Yi Duan, Zhifeng Gao

**Affiliations:** Department of Anesthesiology, Beijing Tsinghua Changgung Hospital, School of Clinical Medicine, Tsinghua Medicine, Tsinghua University, Beijing 102218, China; hhys02463@btch.edu.cn (H.H.); zzs01249@btch.edu.cn (Z.Z.)

**Keywords:** triglyceride-glucose index, arterial stiffness, blood pressure variability, perioperative medicine, estimated pulse wave velocity

## Abstract

**Background:** Intraoperative blood pressure variability (BPV) is associated with adverse perioperative outcomes, although the prognostic meaning of variability itself, once blood pressure level is accounted for, remains uncertain. Preoperative metabolic indicators have not been examined in relation to intraoperative BPV. The triglyceride-glucose (TyG) index, a surrogate marker of insulin resistance, is associated with arterial stiffness in community populations. We examined whether the preoperative TyG index is associated with intraoperative BPV in surgical patients, and whether an estimated pulse wave velocity (ePWV) surrogate statistically accounts for part of this association. **Methods:** This single-center retrospective cohort study included 7081 adults who underwent elective major abdominal surgery under general anesthesia for at least 120 min at Beijing Tsinghua Changgung Hospital between January 2016 and December 2022. The primary outcome was the coefficient of variation of intraoperative mean arterial pressure (CV-MAP). ePWV was calculated from age and preoperative mean arterial pressure (MAP) and is therefore a surrogate rather than a direct measure of arterial stiffness. Multivariable linear regression assessed the TyG-CV-MAP association; models excluding and including age are reported as co-primary analyses. Exploratory mediation analysis using the Baron-Kenny framework with 5000 bootstrap iterations estimated the proportion of the association statistically accounted for by ePWV. Subgroup analyses tested age as an effect modifier, and a sensitivity model additionally adjusted for antihypertensive and antidiabetic medication use. **Results:** After adjustment for sex, body mass index, comorbidities, surgical duration, American Society of Anesthesiologists physical status, and surgery type, each unit increase in the TyG index was associated with a 0.0128-unit increase in CV-MAP (95% confidence interval [CI], 0.0097 to 0.0158; *p* < 0.001); with age additionally included, the estimate was 0.0086 (95% CI, 0.0056 to 0.0117; *p* < 0.001). In absolute terms, each unit increase in TyG corresponded to a 1.074 mmHg higher standard deviation of intraoperative MAP (MAP-SD; 95% CI, 0.842 to 1.305), against a cohort mean MAP-SD of 12.0 mmHg. ePWV statistically accounted for approximately one quarter to one third of the total effect (indirect effect, 0.0038; bootstrap 95% CI, 0.0030 to 0.0046). The association was present in patients younger than 65 years (β = 0.0132, *p* < 0.001), with no evidence of an association in those aged 65 years or older (β = 0.0008, 95% CI, −0.0054 to 0.0071; *p* = 0.79; interaction β = −0.0124, 95% CI, −0.0187 to −0.0061; *p* < 0.001). Sensitivity analyses supported the robustness of these findings, including a model adjusted for antihypertensive and antidiabetic medication use (β = 0.0128, 95% CI, 0.0098 to 0.0159; *p* < 0.001). **Conclusions:** The preoperative TyG index was independently associated with greater intraoperative BPV in major abdominal surgery, with the association present in patients younger than 65 years but not in older patients. An ePWV-based surrogate statistically accounted for approximately one quarter to one third of this association; because ePWV is derived from age and blood pressure rather than measured directly, this proportion may reflect the structure of the surrogate rather than a causal arterial-stiffness pathway. The clinical significance remains uncertain given the modest incremental predictive contribution and the absence of clinically anchored endpoints; prospective validation with directly measured pulse wave velocity is required.

## 1. Introduction

Intraoperative hemodynamic instability is relevant to perioperative patient safety. Intraoperative hypotension is independently associated with postoperative myocardial injury, acute kidney injury, and increased mortality [1,2], whereas the prognostic meaning of blood pressure variability itself, once blood pressure level is accounted for, is less well established [3]. Perioperative hemodynamic management has nonetheless become a focus of recent consensus guidelines [4]. Current preoperative risk assessment tools, including the ASA physical status classification and the revised cardiac risk index (RCRI), reflect comorbidity burden and offer limited evaluation of vascular function [5,6]. Machine learning models built on intraoperative real-time data show predictive capacity [7,8] but cannot provide information before the operation begins. Preoperative metabolic indicators may offer an alternative perspective.

The triglyceride–glucose (TyG) index is a surrogate marker of insulin resistance that correlates well with the hyperinsulinemic–euglycemic clamp [9]. Insulin resistance participates in cardiometabolic disease progression through endothelial dysfunction, chronic low-grade inflammation, and vascular wall remodeling [10]. Large epidemiological studies have confirmed independent associations between the TyG index and atherosclerosis [11,12], type 2 diabetes [13], and arterial stiffness [14,15,16]. The index requires only fasting glucose and triglyceride levels, both of which are routinely available before surgery. These features make it a candidate indicator for perioperative hemodynamic risk stratification.

If the TyG index is related to intraoperative blood pressure stability, arterial stiffness may represent the connecting link. Stiffened arteries lose Windkessel buffering capacity and exhibit augmented pulse wave reflection; at the same time, baroreflex sensitivity declines, and these changes amplify blood pressure fluctuations during the hemodynamic perturbations induced by general anesthesia [17,18]. A recent ambulatory blood pressure study linked the coexistence of elevated TyG and arterial load to greater short-term blood pressure variability [19], whereas longitudinal studies have associated TyG with incident hypertension [20]. A meta-analysis confirmed a dose-response relationship between TyG and arterial stiffness [21]. Preoperative arterial stiffness has been examined as a predictor of hypotension during anesthesia induction [22], but whether metabolic abnormality, vascular stiffening, and intraoperative blood pressure variability are associated in surgical patients has not been directly tested. Estimated pulse wave velocity (ePWV), derived from age and mean arterial pressure alone [23], is suitable for testing this hypothesis in retrospective cohorts that lack direct pulse wave velocity (PWV) measurements.

This study, based on a retrospective cohort of patients undergoing major abdominal surgery, examined three questions: first, whether an elevated preoperative TyG index is independently associated with greater intraoperative BPV; second, what proportion of this association is statistically accounted for by an ePWV-based surrogate; and third, whether age modifies the association. Prior work suggests that the TyG–arterial stiffness relationship is stronger in younger populations, and we hypothesized that the association would be more apparent in patients younger than 65 years.

## 2. Materials and Methods

### 2.1. Study Design and Population

This single-center retrospective cohort study was conducted at Beijing Tsinghua Changgung Hospital. Electronic medical records were extracted from the hospital information system (HIS) and the anesthesia information management system (AIMS) for surgical patients between January 2016 and December 2022. The study protocol was approved by the Medical Ethics Committee of Beijing Tsinghua Changgung Hospital on 4 August 2022 (approval number: 22232-4-02). All research procedures, including database extraction, de-identification, and statistical analysis, were performed after approval was granted; no patient was enrolled prospectively, and no study-specific intervention or data collection was applied. Because the analysis relied solely on de-identified records generated during routine perioperative care, the requirement for written informed consent was waived. The study was reported in accordance with the STROBE guidelines [24].

**Figure 1:**
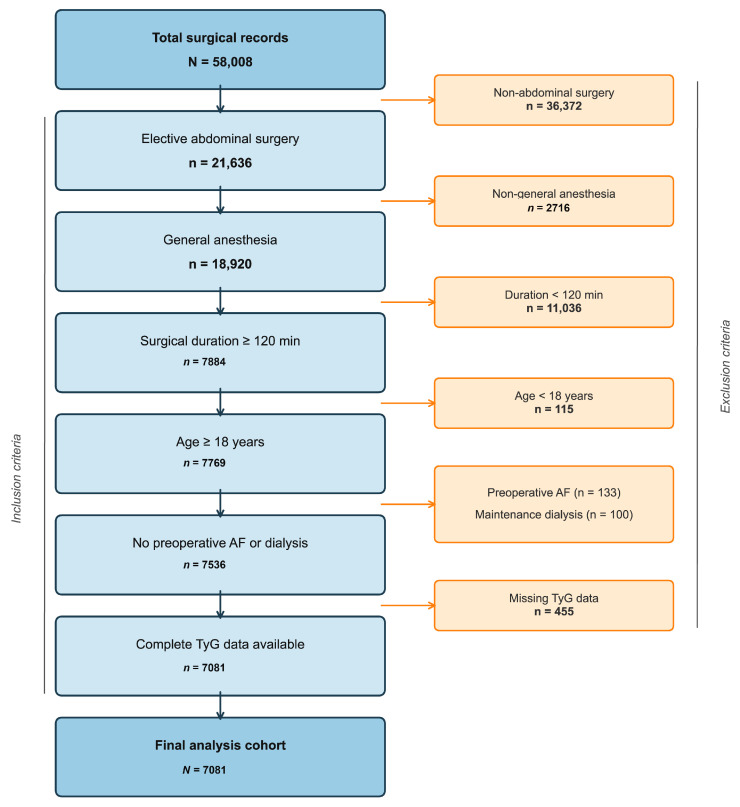
Patient selection flowchart. From 58,008 surgical records at Beijing Tsinghua Changgung Hospital between January 2016 and December 2022, sequential application of inclusion and exclusion criteria yielded a final cohort of 7081 patients. Dark blue boxes mark the initial and final cohorts, light blue boxes mark intermediate eligible cohorts, and orange boxes indicate excluded populations with reasons and counts. AF, atrial fibrillation; TyG, triglyceride-glucose.

The study flow is shown in Figure 1. A total of 58,008 surgical records were identified during the study period. Patients were included if they underwent elective abdominal surgery (hepatobiliary, gastrointestinal, urological, gynecological, or other abdominal surgery), received general anesthesia, had a surgical duration of 120 min or longer, were aged 18 years or older, and had complete preoperative fasting triglyceride and glucose data. Patients with preoperative atrial fibrillation or maintenance dialysis were excluded. The final cohort comprised 7081 patients.

### 2.2. Exposure and Mediator

The TyG index was calculated as ln[fasting triglycerides (mg/dL) × fasting glucose (mg/dL)/2] [9], using laboratory values obtained within 24 h before surgery. The laboratory values reported in mmol/L were converted to mg/dL before calculation (triglycerides: mg/dL = mmol/L × 88.57; glucose: mg/dL = mmol/L × 18.02). TyG was analyzed as both a continuous variable and in quartile groups (Q1 through Q4).

An ePWV-based surrogate of arterial stiffness was computed using the formula from the Reference Values for Arterial Stiffness Collaboration [23]: ePWV = 9.587 − 0.402 × age + 4.560 × 10^−3^ × age^2^ − 2.621 × 10^−5^ × age^2^ × MAP + 3.176 × 10^−3^ × age × MAP − 1.832 × 10^−2^ × MAP, where age is in years and MAP is preoperative mean arterial pressure in mmHg, calculated as diastolic blood pressure + (systolic blood pressure − diastolic blood pressure)/3. Because the formula contains only age and MAP, ePWV reflects the age- and pressure-dependent component of arterial stiffness and is not a direct measurement; carotid-femoral PWV was not available in this cohort. The formula has shown acceptable correlation with carotid–femoral PWV in multiple populations [23]. Because age is the primary determinant of the ePWV formula, models excluding and including age are reported as co-primary analyses (Section 3.4).

### 2.3. Outcomes

The primary outcome was the coefficient of variation of intraoperative MAP (CV-MAP), defined as the standard deviation divided by the mean of sequential intraoperative MAP measurements, consistent with our previous work [7]. Intraoperative MAP was recorded automatically by AIMS at 5 min intervals; invasive arterial monitoring values were used when available, and noninvasive cuff values were used otherwise. Each patient was monitored by a single method throughout: invasive arterial monitoring in 5523 patients (78.0%) and noninvasive cuff measurement in 1558 (22.0%). The recording window extended from anesthesia induction to the end of surgery. Secondary outcomes included MAP standard deviation (MAP-SD), mean pulse pressure (PP-mean), and vasopressor requirement (intraoperative norepinephrine use). CV-MAP at or above the 75th percentile, MAP-SD at or above the 75th percentile, and vasopressor use were also analyzed as binary outcomes. These measures characterize blood pressure variability and vasopressor exposure; they are not validated markers of clinically consequential hemodynamic instability.

### 2.4. Statistical Analysis

Baseline characteristics were compared across TyG quartile groups using ANOVA or the Kruskal–Wallis test for continuous variables and the chi-square test for categorical variables. Trend *p* values were obtained from linear regression with the quartile rank as an ordinal predictor.

The association between TyG and ePWV was evaluated using three progressively adjusted linear regression models: Model 1 adjusted for sex and BMI, Model 2 added hypertension, diabetes, and smoking, and Model 3 added coronary heart disease, heart rate, hemoglobin, albumin, and creatinine. The dose–response relationship was assessed by restricted cubic spline (RCS) regression with knots at the 5th, 35th, 65th, and 95th percentiles of TyG. Multicollinearity was assessed with variance inflation factors (VIF). For multi-category variables, the generalized VIF raised to the power 1/(2 × df) was used, so that indicator sets and single-degree-of-freedom terms are evaluated on a comparable scale; the threshold was 2.24, equivalent to a VIF of 5.

The associations of TyG and ePWV with intraoperative blood pressure variability were analyzed with adjustment for sex, BMI, hypertension, diabetes, surgical duration, ASA class, and surgery type (primary analysis model). The incremental contribution of TyG was measured as the change in R^2^ (ΔR^2^) before and after adding TyG to the base model; for binary outcomes, the change in area under the receiver operating characteristic curve (ΔAUC) was reported (Supplementary Table S6).

Mediation analysis used the Baron–Kenny framework [25] with ePWV as the mediator and CV-MAP and MAP-SD as outcomes. All mediation models were adjusted for the primary analysis covariates (sex, BMI, hypertension, diabetes, surgical duration, ASA class, and surgery type) rather than the fully adjusted Model 3 covariates, because the additional variables in Model 3 (heart rate, hemoglobin, albumin, and creatinine) are potential mediator-outcome confounders that could bias the indirect effect estimate. The indirect effect and its 95% confidence interval were estimated by 5000 nonparametric bootstrap iterations, and the mediated proportion was calculated as the ratio of the indirect effect to the total effect. A counterfactual framework analysis was conducted in parallel [26], reporting the average causal mediation effect (ACME) and the average direct effect (ADE). An exposure–mediator interaction term (TyG × ePWV) was included in the outcome model to test for effect modification (Supplementary Table S4).

Because ePWV incorporates preoperative MAP and CV-MAP is derived from intraoperative MAP, mathematical coupling is possible. Three sensitivity analyses addressed this concern: Pearson correlation coefficients between preoperative MAP and intraoperative variables were calculated; mediation analysis was repeated using residualized ePWV (ePWV regressed on preoperative MAP); and mediation analysis was repeated using age-only ePWV (MAP fixed at the cohort mean, retaining only age-related variation) (Supplementary Table S2).

Effect modification was assessed by adding a TyG × age group interaction term to the regression model. Stratified analyses were performed by age (<65 vs. ≥65 years), sex, diabetes, hypertension, BMI (<25 vs. ≥25 kg/m^2^), and surgery type. The age interaction was interpreted from the interaction estimate and its confidence interval. Two alternative explanations for the attenuated effect in older patients were examined: TyG distributions were compared between age groups to rule out restricted range, and models were additionally adjusted for beta-blocker and statin use to evaluate medication confounding (Supplementary Table S8). Antihypertensive and antidiabetic medication use were ascertained from the preoperative medication reconciliation record and coded as binary variables. Both were identified by matching recorded drug names against a prespecified list. The antihypertensive list corresponded to the Anatomical Therapeutic Chemical (ATC) classes C02, C03, C07, C08, and C09, and therefore includes beta-blockers; the antidiabetic list corresponded to classes A10A (insulins and analogues) and A10B (non-insulin glucose-lowering agents). A further sensitivity model included both medication variables alongside the primary covariates, fitted for CV-MAP and MAP-SD with and without age, to evaluate whether pharmacologic treatment of hypertension or diabetes explained the association (Supplementary Table S9). Other sensitivity analyses included restriction to each age group, exclusion of patients with diabetes or hypertension, exclusion of surgical duration exceeding 480 min, logistic regression with CV-MAP at or above the 75th percentile, and categorical exposure analysis comparing TyG Q4 with Q1 (Supplementary Table S7).

All analyses were performed in R version 4.3.1 (R Foundation for Statistical Computing, Vienna, Austria) using the rms, mediation, and tableone packages. All tests were two-sided with α = 0.05.

## 3. Results

### 3.1. Study Population

The study flow is shown in Figure 1. From 58,008 surgical records, sequential exclusion of non-abdominal surgery (n = 36,372), non-general anesthesia (n = 2716), surgical duration less than 120 min (n = 11,036), age less than 18 years (n = 115), preoperative atrial fibrillation (n = 133), maintenance dialysis (n = 100), and missing TyG data (n = 455) yielded a final cohort of 7081 patients.

Baseline characteristics by TyG quartile are shown in Table 1. The cohort had a mean age of 54.4 ± 15.8 years, 49.6% were male, mean BMI was 26.9 ± 6.2 kg/m^2^, mean TyG index was 9.0 ± 0.5, mean preoperative MAP (calculated from SBP 128.7 ± 15.0 and DBP 70.2 ± 10.0 mmHg) was 89.7 mmHg, and mean ePWV was 8.7 ± 2.5 m/s. Across ascending TyG quartiles, age increased (Q1: 51.3 years vs. Q4: 57.4 years, *p*-trend < 0.001), the prevalence of metabolic comorbidities rose (hypertension: Q1 27.7% vs. Q4 39.3%; diabetes: Q1 8.4% vs. Q4 50.1%), ePWV increased stepwise (Q1: 8.2 m/s vs. Q4: 9.1 m/s, *p*-trend < 0.001), and statin use (Q1: 10.0% vs. Q4: 22.2%) and beta-blocker use (Q1: 7.7% vs. Q4: 13.7%) also increased. Smoking prevalence, surgical duration, and the distribution of surgery types did not differ across groups (all *p* > 0.05). Antihypertensive medication was used by 2282 patients (32.2%) and antidiabetic medication by 1599 (22.6%), both increasing across ascending TyG quartiles (*p*-trend < 0.001). Intraoperative blood pressure was recorded by invasive arterial monitoring in 5523 patients (78.0%) and by noninvasive cuff measurement in 1558 (22.0%).

### 3.2. TyG Index and ePWV

Multivariable regression results for TyG and ePWV are presented in Table 2. TyG was independently and positively associated with ePWV across all three progressively adjusted models: Model 1 (β = 0.677, 95% CI 0.572 to 0.782, *p* < 0.001), Model 2 (β = 0.640, 95% CI 0.532 to 0.749, *p* < 0.001), and Model 3 (β = 0.643, 95% CI 0.535 to 0.751, *p* < 0.001). Slight attenuation from Model 1 to Model 2 suggested partial confounding by hypertension and diabetes. The full model R^2^ was 0.114, and all VIF values were below 1.20.

**Figure 2:**
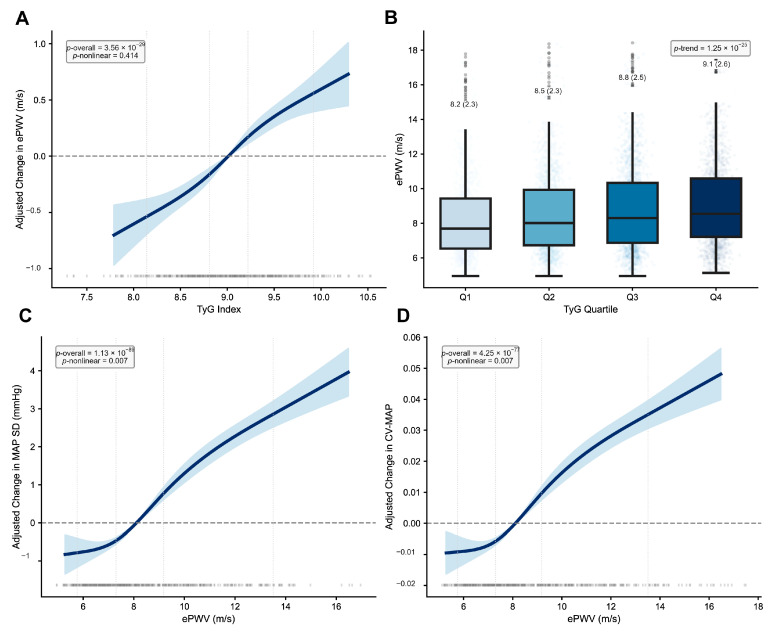
Associations of the TyG index with ePWV and of ePWV with intraoperative blood pressure variability. (**A**) Restricted cubic spline curve for the TyG index and ePWV, adjusted for sex, BMI, hypertension, diabetes, smoking, coronary heart disease, heart rate, hemoglobin, albumin, and creatinine (Model 3). Knots were placed at the 5th, 35th, 65th, and 95th percentiles. The solid line represents the adjusted change in ePWV and the shaded area represents the 95% confidence interval. The reference value was set at the median TyG index. *p*-overall < 0.001; *p*-nonlinear = 0.41. (**B**) Box plots of ePWV by TyG quartile. Values above each box indicate the mean (SD) in m/s. Scattered points represent individual observations. *p*-trend < 0.001. (**C**) Restricted cubic spline curve for ePWV and the standard deviation of intraoperative mean arterial pressure (MAP-SD). *p*-overall < 0.001; *p*-nonlinear = 0.0070. (**D**) Restricted cubic spline curve for ePWV and the coefficient of variation of intraoperative mean arterial pressure (CV-MAP). *p*-overall < 0.001; *p*-nonlinear = 0.0070. Models in (**C**,**D**) were adjusted for sex, BMI, hypertension, diabetes, surgical duration, ASA class, and surgery type. Both associations were positive with mild nonlinearity, showing a relatively flat effect below 8 m/s and accelerating at higher ePWV values. BMI, body mass index; CV-MAP, coefficient of variation of mean arterial pressure; ePWV, estimated pulse wave velocity; MAP-SD, standard deviation of mean arterial pressure; TyG, triglyceride–glucose.

Quartile analysis showed a dose–response relationship: compared with Q1, ePWV increased in Q2 (β = 0.28, *p* < 0.001), Q3 (β = 0.56, *p* < 0.001), and Q4 (β = 0.84, *p* < 0.001; *p*-trend < 0.001). The RCS curve (Figure 2A) showed a linear positive association between TyG and ePWV (*p*-overall < 0.001, *p*-nonlinear = 0.41). The box plot (Figure 2B) illustrated the stepwise increase in ePWV across TyG quartiles.

### 3.3. ePWV and Intraoperative Blood Pressure Variability

The associations of ePWV with intraoperative blood pressure variability are shown in Table 3. Each 1 m/s increase in ePWV was associated with a 0.0061-unit increase in CV-MAP (95% CI 0.0055 to 0.0068, *p* < 0.001), a 0.50 mmHg increase in MAP-SD (95% CI 0.46 to 0.55, *p* < 0.001), and a 0.55 mmHg increase in PP–mean (95% CI 0.42 to 0.67, *p* < 0.001). The RCS curves (Figure 2C,D) showed positive associations with mild nonlinearity (*p*-nonlinear = 0.0070): the effect was relatively flat below an ePWV of 8 m/s and accelerated at higher values, consistent with a threshold pattern in this age- and pressure-derived surrogate.

### 3.4. TyG Index and Intraoperative Blood Pressure Variability

The associations of TyG with intraoperative outcomes are shown in Table 3. TyG was independently associated with CV-MAP (β = 0.0128, 95% CI 0.0097 to 0.0158, *p* < 0.001), MAP-SD (β = 1.074, 95% CI 0.842 to 1.305, *p* < 0.001), and PP-mean (β = 0.880, 95% CI 0.293 to 1.468, *p* = 0.0030). Model R^2^ was 0.031, with TyG contributing ΔR^2^ = 0.009 (Supplementary Table S6).

In logistic regression (Table 3), each unit increase in TyG was associated with a 24% increase in the odds of vasopressor use (OR 1.24, 95% CI 1.05 to 1.47, *p* = 0.011), a 37% increase in the odds of CV-MAP at or above the 75th percentile (OR 1.37, 95% CI 1.23 to 1.53, *p* < 0.001), and a 44% increase in the odds of MAP-SD at or above the 75th percentile (OR 1.44, 95% CI 1.29 to 1.61, *p* < 0.001). After additional adjustment for age (co-primary model), the association with vasopressor use was no longer significant (OR 1.16, 95% CI 0.97 to 1.37, *p* = 0.098), whereas the associations with CV-MAP and MAP-SD at or above the 75th percentile persisted (OR 1.23, 95% CI 1.10 to 1.38 and OR 1.30, 95% CI 1.16 to 1.45, respectively; both *p* < 0.001). Discrimination improved modestly after adding TyG: ΔAUC ranged from 0.007 to 0.018 (Supplementary Table S6). MAP-SD, norepinephrine use, and CV-MAP each increased across ascending TyG quartiles (Supplementary Figure S2).

Models excluding and including age are reported as co-primary analyses (Table 3 and Table S5). With age included, the TyG effect on CV-MAP was attenuated but persisted (β = 0.0086, 95% CI 0.0056 to 0.0117, *p* < 0.001), and model R^2^ rose from 0.031 to 0.072. The effect on MAP-SD was also preserved (β = 0.739, 95% CI 0.511 to 0.968, *p* < 0.001), while the effect on PP–mean became marginally non-significant (β = 0.552, 95% CI −0.041 to 1.144, *p* = 0.068). In absolute terms, each unit increase in TyG corresponded to a 1.074 mmHg higher MAP-SD (95% CI 0.842 to 1.305) in the age-excluded model against a cohort mean MAP-SD of 12.0 mmHg. A sensitivity model additionally adjusted for antihypertensive and antidiabetic medication use left the TyG estimates essentially unchanged (Supplementary Table S9), indicating that the association is not explained by these comorbidities or their pharmacologic treatment.

### 3.5. Mediation Analysis

**Figure 3:**
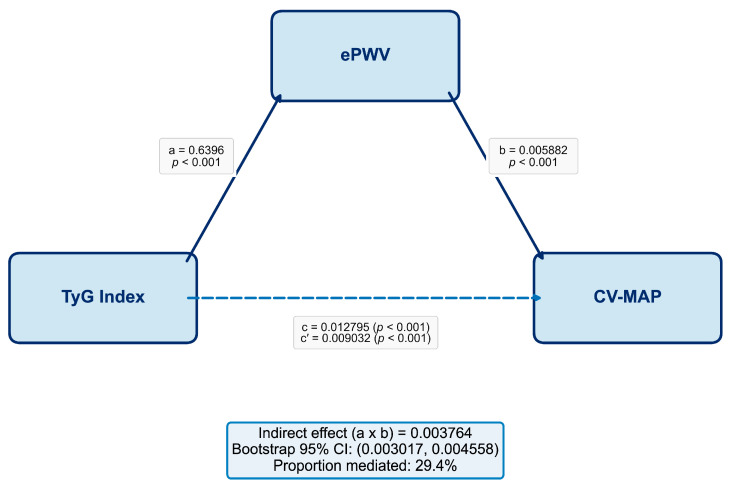
Mediation analysis path diagram showing the role of ePWV in the association between the TyG index and CV-MAP. Solid arrows denote the a and b paths through ePWV. The dashed arrow denotes the TyG to CV-MAP path, labeled with the total effect c and direct effect c′.

Mediation analysis results are shown in Table 4 and Figure 3. For the CV-MAP outcome, the a path (TyG → ePWV, adjusted for the primary analysis covariates) was β = 0.640 (*p* < 0.001), the b path (ePWV → CV-MAP, adjusted for TyG) was β = 0.0059 (*p* < 0.001), the total effect (c path) was β = 0.0128, the direct effect (c′ path) was β = 0.0090, and the indirect effect (a × b) was 0.0038 (bootstrap 95% CI 0.0030 to 0.0046), corresponding to a mediated proportion of 29.4%. For the MAP-SD outcome, the indirect effect was 0.308 (bootstrap 95% CI 0.247 to 0.372), with a mediated proportion of 28.7%.

The a path represents the effect of TyG on ePWV (β = 0.640, *p* < 0.001), and the b path represents the effect of ePWV on CV-MAP adjusted for TyG (β = 0.0059, *p* < 0.001). The solid arrows show the a and b paths through ePWV, whereas the dashed TyG to CV-MAP arrow is labeled with the total effect (c = 0.0128, *p* < 0.001) and the direct effect (c′ = 0.0090, *p* < 0.001). The indirect effect (a × b) was 0.0038 (bootstrap 95% CI, 0.0030 to 0.0046), and the mediated proportion was 29.4%. All paths were adjusted for sex, BMI, hypertension, diabetes, surgical duration, ASA physical status, and surgery type, with 5000 bootstrap iterations. CV-MAP, coefficient of variation of mean arterial pressure; ePWV, estimated pulse wave velocity; TyG, triglyceride-glucose.

The TyG × ePWV interaction term reached significance (CV-MAP: β = −0.0021, *p* < 0.001; MAP-SD: β = −0.167, *p* < 0.001) (Supplementary Table S4), indicating that the association between TyG and BPV weakened as ePWV increased, a pattern consistent with that observed across age subgroups. Counterfactual framework analysis yielded consistent estimates (Supplementary Table S3).

### 3.6. Sensitivity Analyses for Mathematical Coupling

Preoperative MAP was weakly correlated with intraoperative MAP mean (r = 0.120), MAP-SD (r = 0.127), and CV-MAP (r = 0.095; all *p* < 0.001) (Supplementary Table S2). When residualized ePWV (R^2^ = 0.385 for the regression of ePWV on preoperative MAP) replaced raw ePWV in the mediation model, the indirect effect remained significant (CV-MAP: 0.0032, 95% CI 0.0025 to 0.0039, mediated proportion 24.8%; MAP-SD: 0.252, 95% CI 0.195 to 0.311, mediated proportion 23.5%), with a decrease of approximately five percentage points compared with the primary analysis. When MAP was fixed at the cohort mean of 89.7 mmHg (age-only ePWV), the proportion statistically accounted for increased (CV-MAP: 30.5%; MAP-SD: 29.4%), suggesting that this proportion tracks the age-related component of the surrogate rather than mathematical coupling through the MAP component.

### 3.7. Subgroup Analysis and Effect Modification

Subgroup analysis results are shown in Table 5 and Supplementary Figure S1. Age modified the TyG-CV-MAP association (interaction β = −0.0124, 95% CI −0.0187 to −0.0061, *p* < 0.001): the association was present in patients younger than 65 years (β = 0.0132, 95% CI 0.0098 to 0.0166, *p* < 0.001), with no evidence of an association in those aged 65 years or older (β = 0.0008, 95% CI −0.0054 to 0.0071, *p* = 0.79).

TyG distributions were similar in both age groups, and additional adjustment for beta-blocker and statin use did not alter the results in either group (Supplementary Table S8), making restricted range and medication confounding unlikely explanations. The older-group estimate is reported as no evidence of an association rather than as evidence of no effect, and the age interaction is interpreted from the interaction estimate and its confidence interval as a hypothesis-generating finding.

Sex (*p*-interaction = 0.96), diabetes (*p*-interaction = 0.18), hypertension (*p*-interaction = 0.28), and BMI (*p*-interaction = 0.32) did not modify the association. The TyG-CV-MAP association was directionally consistent across all four surgery types (β ranging from 0.0100 to 0.0186, all *p* < 0.05).

Additional sensitivity analyses (Supplementary Table S7) confirmed the robustness of the primary finding: the association persisted after excluding patients with diabetes (β = 0.0115, *p* < 0.001), excluding those with hypertension (β = 0.0138, *p* < 0.001), and excluding procedures lasting more than 480 min (β = 0.0130, *p* < 0.001). Logistic regression for CV-MAP at or above the 75th percentile (OR 1.37, *p* < 0.001) and categorical analysis of TyG Q4 versus Q1 (β = 0.0177, *p* < 0.001) produced consistent results.

## 4. Discussion

This study found that the preoperative TyG index was independently associated with intraoperative CV-MAP in a dose-response manner in patients undergoing major abdominal surgery. An ePWV-based surrogate statistically accounted for approximately one quarter to one third of this association, and the association was present in patients younger than 65 years, with no evidence of an association in older patients. The TyG index has been used for cardiovascular risk assessment in community populations; the present study examines its association with perioperative BPV. A recent ambulatory blood pressure study linked the coexistence of elevated TyG and arterial load to blood pressure variability [19], whereas other studies associated TyG with hypertension [20] and arterial stiffness [14,15]. Perioperative metabolic research has focused on glycemic control [27], and TyG has not been examined in the surgical setting. Our group previously used machine learning to identify predictors of intraoperative BPV [7,8]; the present study examines a preoperative metabolic marker.

The incremental contribution of TyG to BPV prediction was modest: adding TyG increased model R^2^ by only 0.009, and the AUC for high CV-MAP increased by 0.014. In the full model (Supplementary Table S1), hypertension was the only covariate other than TyG and surgical duration that reached significance (β = 0.0085), and TyG had a larger effect (β = 0.0128). CV-MAP is determined largely by intraoperative factors (anesthetic depth, surgical manipulation, volume shifts) that cannot be captured by preoperative variables; the 3.1% explained variance is consistent with the expected contribution of a single preoperative metabolic marker. In absolute terms, each unit increase in TyG corresponded to a 1.074 mmHg higher MAP-SD (95% CI 0.842 to 1.305) against a cohort mean MAP-SD of 12.0 mmHg. Placing this magnitude in clinical context requires caution. In the largest analysis of intraoperative MAP variability and 30-day mortality, Mascha et al. [3] found that time-weighted average MAP, not variability, was the dominant correlate of mortality, which more than tripled as time-weighted average MAP fell from 80 to 50 mmHg; variability itself was only weakly related after adjustment for MAP level (*p* = 0.033), and lower rather than higher variability tracked with higher mortality. Subsequent outcome analyses in noncardiac surgery have likewise centered on the depth and duration of hypotension rather than on its variability [28]. No threshold of intraoperative MAP variability with established prognostic meaning is therefore available, and we do not claim that the TyG-attributable increment crosses one. We present the association as a statistical association whose physiological basis and clinical consequences remain to be established, and we make no claim that TyG improves perioperative risk stratification; formal reclassification and decision-curve analyses would require hard clinical endpoints that this cohort does not contain. With age included as a co-primary model, the TyG effect was attenuated by approximately one third (β from 0.0128 to 0.0086) but persisted (*p* < 0.001), indicating that TyG carries metabolic information beyond age. PP–mean became marginally non-significant after age adjustment (*p* = 0.068), consistent with the collinearity between age and pulse pressure. The TyG effect was robust after excluding patients with diabetes or hypertension, and after adjustment for antihypertensive and antidiabetic medication use (β = 0.0128, 95% CI 0.0098 to 0.0159, *p* < 0.001), indicating that the association is not explained by these comorbidities or their pharmacologic treatment. Possible mechanisms by which insulin resistance affects intraoperative hemodynamics include reduced endothelial nitric oxide bioavailability [29], impaired baroreflex function [30], and inflammation-altered vascular reactivity [31]; these were not measured in the present study, and mechanistic inference requires prospective investigation.

The Baron–Kenny method estimated that ePWV statistically accounted for 29.4% of the TyG effect on CV-MAP and 28.7% on MAP-SD; the counterfactual framework produced consistent results (ACME = 0.0038, proportion 29.4%). The a path coefficient in the mediation model (0.640) was estimated with the primary analysis covariate set rather than the fully adjusted Model 3 covariate set (0.643), which accounts for the numerical difference between Table 2 and Table 4. He et al. [32] reported that ePWV accounted for 42.7% of the association between TyG-ABSI and cardiovascular mortality in NHANES, a comparable estimate. Because ePWV incorporates preoperative MAP and CV-MAP is derived from intraoperative MAP, mathematical coupling is possible. Preoperative MAP correlated weakly with intraoperative variables (r = 0.095 to 0.127), and residualized ePWV analysis reduced the proportion from 29.4% to 24.8%, with approximately 5 percentage points attributable to shared MAP components. Fixing MAP at the cohort mean and retaining only age variation raised the proportion to 30.5%. That an ePWV term varying only with age accounts for a comparable proportion suggests that the signal may largely reflect the age- and blood-pressure-related structure of the surrogate itself; this reading, rather than mathematical coupling alone, is the more parsimonious interpretation, and it should not be taken as evidence of a causal or modifiable arterial-stiffness mechanism. The estimate is therefore best described as the proportion statistically accounted for by an age- and pressure-derived surrogate, not as evidence of a causal biological pathway.

The TyG × ePWV interaction was significant (β = −0.0021, *p* < 0.001), indicating that the TyG association weakened as ePWV increased. The Baron–Kenny method may produce biased estimates when exposure-mediator interaction exists [26], but the counterfactual proportion was identical (29.4%), suggesting limited impact on the estimate. Approximately 70% of the association was not accounted for by ePWV. Because ePWV is a function of age and MAP, it captures only the age- and pressure-dependent component of arterial stiffness and omits the metabolic and inflammatory component through which TyG may act; directly measured carotid–femoral PWV would capture this component, so the modest proportion accounted for here likely reflects, in part, this structural limitation of the surrogate rather than a genuinely small stiffness pathway. Li et al. [33] reported that lipids and inflammation partially accounted for the TyG–arterial stiffness association; autonomic dysfunction [30], endothelial injury [29], and systemic inflammation [31] may represent parallel pathways. Heart rate variability and inflammatory markers could be included in multiple mediator models in future studies. Age was excluded from one of the two co-primary models because the ePWV formula contains age, and simultaneous adjustment would remove mediator-related variation linked to the exposure [26]; the age-adjusted model is reported alongside it, and the core findings were unchanged.

The TyG-CV-MAP association was present in patients younger than 65 years (β = 0.0132), with no evidence of an association in older patients (β = 0.0008, 95% CI −0.0054 to 0.0071; interaction β = −0.0124, 95% CI −0.0187 to −0.0061, *p* < 0.001). Two alternative explanations were examined. TyG distributions were similar in both groups (SD 0.54 vs. 0.53), making restricted range unlikely, and additional adjustment for beta-blockers and statins did not change the older-group result (β = 0.0009, *p* = 0.79), making medication confounding unlikely. One hypothesis is an arterial-stiffness ceiling: in older patients, age-related stiffening has already elevated baseline stiffness [34] and compliance reserves are reduced, so the incremental stiffness contributed by insulin resistance would have little hemodynamic consequence. Chen et al. [35] reported that age-related arterial and ventricular stiffening increase in parallel. An alternative explanation cannot be excluded: because ePWV is itself a function of age, an ePWV-based analysis cannot distinguish a true arterial-stiffness ceiling from the mathematical structure of the surrogate. The negative TyG × ePWV interaction is consistent with both readings. Separating them would require directly measured carotid–femoral PWV in a prospective design. We therefore present the ceiling account as a hypothesis rather than a demonstrated mechanism.

One implication is that younger patients without overt cardiovascular comorbidities are typically classified as low risk by existing assessment tools, yet those with an elevated TyG index showed greater intraoperative blood pressure variability in this cohort. The TyG index requires only fasting glucose and triglyceride measurements and does not depend on additional equipment. Whether this information should alter intraoperative monitoring or hemodynamic management cannot be determined from a retrospective study without clinical endpoints, and we make no such recommendation here. Whether preoperative metabolic optimization improves intraoperative circulatory stability, and whether TyG-guided management affects postoperative outcomes, require prospective investigation.

This study has several limitations. The single-center retrospective design limits external generalizability. ePWV is a surrogate derived from age and blood pressure, not a direct measure of arterial stiffness; the mediation estimates should be interpreted accordingly, and residual bias from mathematical coupling cannot be fully excluded. Anesthetic technique and depth, fluid and vasopressor management, blood loss, transfusion, and pneumoperitoneum were not consistently recorded in the structured AIMS export; residual confounding by these intraoperative factors cannot be excluded. Monitoring modality differed across patients (78.0% invasive arterial, 22.0% noninvasive cuff), and the two methods may yield different variability estimates [36]. CKD severity was not captured. TyG values depend on fasting duration, which may vary across surgical services; such measurement variability would attenuate the observed associations toward the null. Age-related residual confounding cannot be excluded, although models with and without age are reported as co-primary analyses. Prospective validation with directly measured carotid–femoral PWV and clinically anchored endpoints is required.

## 5. Conclusions

The preoperative TyG index was independently associated with greater intraoperative BPV in major abdominal surgery, with the association present in patients younger than 65 years but not in older patients. An ePWV-based surrogate statistically accounted for approximately one quarter to one third of this association; because ePWV is derived from age and blood pressure rather than measured directly, this proportion may reflect the structure of the surrogate rather than a causal arterial-stiffness pathway. The clinical significance remains uncertain given the modest incremental predictive contribution and the absence of clinically anchored endpoints; prospective validation with directly measured pulse wave velocity is required.

## Figures and Tables

**Table 1:** Baseline characteristics by TyG index quartiles.

Variable	Overall	Q1	Q2	Q3	Q4	*p*	*p*-Trend
N	7081	1775	1796	1771	1739		
Age (years)	54.4 (15.8)	51.3 (16.0)	53.8 (15.4)	55.4 (15.8)	57.4 (15.4)	<0.001	<0.001
Male, n (%)	3509 (49.6)	862 (48.6)	863 (48.1)	903 (51.0)	881 (50.7)	0.20	0.082
BMI (kg/m^2^)	26.9 (6.2)	26.1 (6.0)	26.7 (6.1)	27.1 (6.2)	27.7 (6.2)	<0.001	<0.001
TG (mmol/L) †	1.9 [1.4–2.6]	1.7 [1.2–2.2]	1.8 [1.3–2.5]	2.0 [1.4–2.6]	2.1 [1.6–2.9]	<0.001	<0.001
FPG (mmol/L) †	5.4 [4.7–6.4]	4.7 [4.2–5.5]	5.2 [4.6–5.9]	5.6 [4.9–6.5]	6.3 [5.5–7.4]	<0.001	<0.001
TyG index	9.0 (0.5)	8.3 (0.3)	8.8 (0.1)	9.2 (0.1)	9.7 (0.3)	<0.001	<0.001
ePWV (m/s)	8.7 (2.5)	8.2 (2.3)	8.5 (2.3)	8.8 (2.5)	9.1 (2.6)	<0.001	<0.001
SBP (mmHg)	128.7 (15.0)	126.7 (15.3)	127.9 (14.7)	129.3 (14.9)	130.9 (14.7)	<0.001	<0.001
DBP (mmHg)	70.2 (10.0)	69.1 (10.1)	69.9 (10.1)	70.8 (10.0)	71.2 (9.9)	<0.001	<0.001
MAP pre (mmHg)	89.7	88.3	89.3	90.3	91.1	<0.001	<0.001
Hypertension, n (%)	2352 (33.2)	491 (27.7)	564 (31.4)	613 (34.6)	684 (39.3)	<0.001	<0.001
Diabetes, n (%)	1826 (25.8)	149 (8.4)	312 (17.4)	494 (27.9)	871 (50.1)	<0.001	<0.001
CHD, n (%)	581 (8.2)	117 (6.6)	128 (7.1)	141 (8.0)	195 (11.2)	<0.001	<0.001
Smoking, n (%)	1752 (24.7)	439 (24.7)	439 (24.4)	442 (25.0)	432 (24.8)	0.99	0.85
Beta-blocker, n (%)	736 (10.4)	136 (7.7)	179 (10.0)	182 (10.3)	239 (13.7)	<0.001	<0.001
Statin, n (%)	1055 (14.9)	177 (10.0)	236 (13.1)	256 (14.5)	386 (22.2)	<0.001	<0.001
Antihypertensive, n (%)	2282 (32.2)	489 (27.5)	580 (32.3)	566 (32.0)	647 (37.2)	<0.001	<0.001
Antidiabetic, n (%)	1599 (22.6)	150 (8.5)	284 (15.8)	416 (23.5)	749 (43.1)	<0.001	<0.001
Duration (min) †	200 [152–262]	196 [151–260]	203 [150–265]	199 [154–260]	202 [153–266]	0.42	0.26
MAP mean (mmHg)	79.1 (8.1)	78.6 (8.1)	78.9 (8.2)	79.2 (8.0)	79.5 (7.8)	0.0080	<0.001
MAP-SD (mmHg)	12.0 (5.0)	11.3 (4.9)	11.9 (5.1)	12.1 (5.0)	12.7 (5.1)	<0.001	<0.001
CV-MAP	0.152 (0.068)	0.145 (0.067)	0.151 (0.069)	0.154 (0.068)	0.161 (0.069)	<0.001	<0.001
PP mean (mmHg)	45.0 (12.7)	44.4 (12.4)	44.7 (12.8)	45.3 (12.8)	45.5 (12.6)	0.030	0.0030
NE use, n (%)	597 (8.4)	125 (7.0)	153 (8.5)	138 (7.8)	181 (10.4)	0.0030	0.0020

Continuous variables are presented as mean (SD) or, where marked by †, median [IQR], except for preoperative MAP, for which group means are presented. Categorical variables are presented as n (%). CV-MAP is reported to three decimal places. Preoperative MAP was calculated as DBP + (SBP − DBP)/3. *p* values were derived from ANOVA, the Kruskal-Wallis test, or the chi-square test. *p*-trend values were derived from linear regression with quartile rank as an ordinal predictor, except for antihypertensive and antidiabetic medication use, for which group comparisons used the Pearson chi-square test and trend tests used the Cochran-Armitage test. Beta-blockers are a subset of the antihypertensive category (ATC C07) and are not counted separately. BMI, body mass index; CHD, coronary heart disease; CV-MAP, coefficient of variation of mean arterial pressure; DBP, diastolic blood pressure; ePWV, estimated pulse wave velocity; FPG, fasting plasma glucose; MAP, mean arterial pressure; MAP-SD, standard deviation of mean arterial pressure; NE, norepinephrine; PP, pulse pressure; SBP, systolic blood pressure; TG, triglycerides; TyG, triglyceride-glucose.

**Table 2:** Association between TyG index and ePWV: progressively adjusted models.

Analysis	Model 1 β (95% CI)	Model 2 β (95% CI)	Model 3 β (95% CI)
TyG (continuous)	0.677 (0.572, 0.782)	0.640 (0.532, 0.749)	0.643 (0.535, 0.751)
Q2 vs. Q1	NA	NA	0.277 (0.124, 0.430)
Q3 vs. Q1	NA	NA	0.559 (0.403, 0.714)
Q4 vs. Q1	NA	NA	0.838 (0.674, 1.002)
*p*-trend	NA	NA	<0.001
R^2^	0.022	0.113	0.114

All *p* < 0.001 for continuous TyG. All VIF < 1.20 in this model, which does not include ASA class or surgery type. Age not included to avoid overadjustment (see Methods). Model 1 adjusted for sex and BMI. Model 2 added hypertension, diabetes, and smoking. Model 3 added coronary heart disease, heart rate, hemoglobin, albumin, and creatinine.

**Table 3:** Associations of TyG index and ePWV with intraoperative blood pressure variability.

Outcome	Predictor	Model	β/OR	95% CI	*p*
**Linear regression**					
CV-MAP	ePWV	Without age	0.0061	(0.0055, 0.0068)	<0.001
MAP-SD	ePWV	Without age	0.503	(0.455, 0.552)	<0.001
PP-mean	ePWV	Without age	0.548	(0.423, 0.673)	<0.001
CV-MAP	TyG	Without age	0.0128	(0.0097, 0.0158)	<0.001
CV-MAP	TyG	With age	0.0086	(0.0056, 0.0117)	<0.001
MAP-SD	TyG	Without age	1.074	(0.842, 1.305)	<0.001
MAP-SD	TyG	With age	0.739	(0.511, 0.968)	<0.001
PP-mean	TyG	Without age	0.880	(0.293, 1.468)	0.0030
PP-mean	TyG	With age	0.552	(−0.041, 1.144)	0.068
**Logistic regression**					
NE use	TyG	Without age	1.24	(1.05, 1.47)	0.011
NE use	TyG	With age	1.16	(0.97, 1.37)	0.098
CV-MAP ≥ P75	TyG	Without age	1.37	(1.23, 1.53)	<0.001
CV-MAP ≥ P75	TyG	With age	1.23	(1.10, 1.38)	<0.001
MAP-SD ≥ P75	TyG	Without age	1.44	(1.29, 1.61)	<0.001
MAP-SD ≥ P75	TyG	With age	1.30	(1.16, 1.45)	<0.001

All models adjusted for sex, BMI, hypertension, diabetes, surgical duration, ASA class, and surgery type. Models excluding and including age are reported as co-primary analyses, because the ePWV formula is derived from age and simultaneous adjustment removes exposure-related variation in the surrogate. ePWV models are presented without age for the same reason and cannot be age-adjusted. Linear regression results reported as β; logistic regression results reported as OR. Full model output including R^2^ is given in Supplementary Table S5.

**Table 4:** Mediation analysis of ePWV in the TyG-BPV association.

Analysis	N	β/Effect	95% CI	*p*	% Mediated
**Mediation: CV-MAP**					
Total effect (c)	7081	0.0128	(0.0097, 0.0158)	<0.001	
Direct effect (c′)		0.0090	(0.006, 0.012)	<0.001	
a: TyG → ePWV		0.640	(0.532, 0.748)	<0.001	
b: ePWV → Y|TyG		0.0059	(0.005, 0.007)	<0.001	
Indirect effect (a × b)		0.0038	(0.0030, 0.0046) *	<0.001	29.4%
**Mediation: MAP-SD**					
Total effect (c)	7081	1.074	(0.842, 1.305)	<0.001	
Direct effect (c′)		0.765	(0.538, 0.993)	<0.001	
a: TyG → ePWV		0.640	(0.532, 0.748)	<0.001	
b: ePWV → Y|TyG		0.481	(0.432, 0.530)	<0.001	
Indirect effect (a × b)		0.308	(0.247, 0.372) *	<0.001	28.7%

The a path (TyG → ePWV), b path (ePWV → outcome), and all effects were estimated with the primary analysis covariate set (sex, BMI, hypertension, diabetes, surgical duration, ASA class, and surgery type), not the fully adjusted Model 3 covariate set used in Table 2. This accounts for the difference between the a path coefficient reported here (0.640) and the corresponding Model 3 coefficient in Table 2 (0.643). * 95% CI estimated by 5000 nonparametric bootstrap iterations. Counterfactual framework results are reported in Supplementary Table S3.

**Table 5:** Subgroup analysis and effect modification of the TyG-CV-MAP association.

Analysis	N	β/Effect	95% CI	*p*	*p*-int.
**Subgroup: TyG → CV-MAP**					
Age < 65	5223	0.0132	(0.0098, 0.0166)	<0.001	<0.001
Age ≥ 65	1858	0.0008	(−0.0054, 0.0071)	0.79	
Male	3509	0.0122	(0.0079, 0.0166)	<0.001	0.96
Female	3572	0.0133	(0.0090, 0.0176)	<0.001	
Diabetes: No	5255	0.0115	(0.0080, 0.0151)	<0.001	0.18
Diabetes: Yes	1826	0.0166	(0.0106, 0.0225)	<0.001	
Hypertension: No	4729	0.0138	(0.0101, 0.0175)	<0.001	0.28
Hypertension: Yes	2352	0.0107	(0.0054, 0.0161)	<0.001	
BMI < 25	2765	0.0115	(0.0065, 0.0164)	<0.001	0.32
BMI ≥ 25	4316	0.0134	(0.0096, 0.0173)	<0.001	
Hepatobiliary	2132	0.0101	(0.0045, 0.0157)	<0.001	
Gastrointestinal	1739	0.0129	(0.0066, 0.0191)	<0.001	
Urology	1448	0.0186	(0.0117, 0.0255)	<0.001	
Gynecology	1063	0.0100	(0.0025, 0.0175)	0.0090	

Each subgroup model was adjusted for the primary analysis covariate set, excluding the stratification variable itself. *p*-int., *p* value for interaction. Surgery-type subgroups show the four main categories.

## Data Availability

The data are available from the corresponding authors upon reasonable request. The data are not publicly available because they contain potentially sensitive clinical information and are subject to institutional privacy and ethical restrictions.

## Supplementary Materials

The following supporting information can be downloaded at: https://www.mdpi.com/article/10.3390/jcm15156058/s1, Table S1. Full model coefficients: TyG index and CV-MAP. Table S2. Mathematical coupling sensitivity analyses; Table S3. Counterfactual mediation analysis; Table S4. TyG × ePWV interaction in outcome models; Table S5. TyG effect with and without age adjustment; Table S6. Incremental discrimination of TyG index; Table S7. Sensitivity analyses; Table S8. Age-specific analyses: TyG distribution, post-hoc power, and medication-adjusted results; Table S9. Sensitivity analyses adjusted for antihypertensive and antidiabetic medication use; Figure S1. Forest plot of the TyG index-CV-MAP association by age subgroup; Figure S2. Intraoperative hemodynamic profile by TyG index quartile. (**A**) Violin plot of the standard deviation of intraoperative mean arterial pressure (MAP-SD). *p*-trend < 0.001. (**B**) Bar chart of intraoperative norepinephrine (NE) use rate by quartile. Numbers above each bar indicate the count of NE users and total patients in each group. *p*-trend = 0.0020. (**C**) Box plot of the coefficient of variation of intraoperative mean arterial pressure (CV-MAP). *p*-trend < 0.001. All three hemodynamic indicators increased across ascending TyG quartiles. *p*-trend values were derived from linear regression with quartile rank as an ordinal predictor. CV-MAP, coefficient of variation of mean arterial pressure; MAP-SD, standard deviation of mean arterial pressure; NE, norepinephrine; TyG, triglyceride-glucose.

## Abbreviations

ABSI: a body shape index; ACME: average causal mediation effect; ADE: average direct effect; AIMS: anesthesia information management system; ASA: American Society of Anesthesiologists; ATC: Anatomical Therapeutic Chemical; AUC: area under the receiver operating characteristic curve; BMI: body mass index; BPV: blood pressure variability; CI: confidence interval; CV-MAP: coefficient of variation of mean arterial pressure; ePWV: estimated pulse wave velocity; HIS: hospital information system; MAP: mean arterial pressure; MAP-SD: standard deviation of mean arterial pressure; NHANES: National Health and Nutrition Examination Survey; OR: odds ratio; PP: pulse pressure; PWV: pulse wave velocity; RCRI: revised cardiac risk index; RCS: restricted cubic spline; STROBE: Strengthening the Reporting of Observational Studies in Epidemiology; TyG: triglyceride-glucose; VIF: variance inflation factor.
