# Supplementary material for: Association Between the Preoperative Triglyceride-Glucose Index and Intraoperative Blood Pressure Variability in Major Abdominal Surgery: A Retrospective Cohort Study
Source: J Clin Med. 2026 Aug 4;15(15):6058. doi: 10.3390/jcm15156058 (PMC13467104; doi:10.3390/jcm15156058)
Supplement: Supplementary file 1 [file jcm-15-06058-s001.zip › jcm-4427331-supplementary.pdf]

Supplementary Tables

Supplementary Table S1. Full model coefficients: TyG index and CV-MAP

Variable	β	95% CI	*p*
TyG	0.0128	(0.0097, 0.0158)	<0.001
Sex (Male)	0.0006	(−0.0025, 0.0036)	0.712
BMI	−0.00001	(−0.0003, 0.0002)	0.963
Hypertension	0.0085	(0.0052, 0.0118)	<0.001
Diabetes	−0.0034	(−0.0072, 0.0004)	0.077
Duration	0.00010	(0.00008, 0.00011)	<0.001
ASA II	−0.0020	(−0.0109, 0.0070)	0.669
ASA III	−0.0048	(−0.0138, 0.0043)	0.304
ASA IV	−0.0037	(−0.0154, 0.0080)	0.534

R^2 = 0.031, adjusted R^2 = 0.029, F = 17.34. N = 7081. Surgery type dummy variables omitted (all p > 0.5).

Supplementary Table S2. Mathematical coupling sensitivity analyses

A. Pearson correlation: preoperative MAP vs intraoperative variables

Variable pair	r	*p*	N
MAP pre vs MAP mean	0.120	<0.001	7081
MAP pre vs MAP-SD	0.127	<0.001	7081
MAP pre vs CV-MAP	0.095	<0.001	7081

B. Residualized ePWV mediation (preoperative MAP removed, R^2 = 0.385)

Outcome	Indirect effect	95% CI	*p*	% Mediated
CV-MAP	0.0032	(0.0025, 0.0039)	<0.001	24.8%
MAP-SD	0.252	(0.195, 0.311)	<0.001	23.5%

C. Age-only ePWV mediation (MAP pre fixed at 89.7 mmHg)

Outcome	Indirect effect	95% CI	*p*	% Mediated
CV-MAP	0.0039	(0.0031, 0.0047)	<0.001	30.5%
MAP-SD	0.315	(0.254, 0.380)	<0.001	29.4%

Bootstrap: 5000 iterations.

Supplementary Table S3. Counterfactual mediation analysis

Outcome	ACME	95% CI	ADE	95% CI	% Mediated
CV-MAP	0.0038	(0.0030, 0.0046)	0.0090	(0.0060, 0.0120)	29.4%
MAP-SD	0.307	(0.248, 0.372)	0.766	(0.535, 1.001)	28.6%

ACME, average causal mediation effect; ADE, average direct effect. All p < 0.001. Bootstrap: 5000 iterations.

Supplementary Table S4. TyG × ePWV interaction in outcome models

Outcome	Interaction β	SE	*p*	R^2
CV-MAP	−0.00211	0.000555	<0.001	0.075
MAP-SD	−0.167	0.042	<0.001	0.085

Model: outcome ~ TyG + ePWV + TyG × ePWV + covariates.

Supplementary Table S5. TyG effect with and without age adjustment

Outcome	Model	TyG β	95% CI	*p*	R^2
CV-MAP	Without age	0.0128	(0.0097, 0.0158)	2.3×10^{-16}	0.031
	With age	0.0086	(0.0056, 0.0117)	2.1×10^{-8}	0.072
MAP-SD	Without age	1.074	(0.842, 1.305)	1.2×10^{-19}	0.035
	With age	0.739	(0.511, 0.968)	2.3×10^{-10}	0.081
PP-mean	Without age	0.880	(0.293, 1.468)	0.003	0.012
	With age	0.552	(−0.041, 1.144)	0.068	0.019

Models adjusted for sex, BMI, hypertension, diabetes, surgical duration, ASA class, and surgery type. ePWV was not included in these models to avoid collinearity with age.

Supplementary Table S6. Incremental discrimination of TyG index

A. Incremental R^2 (continuous outcomes)

Outcome	R^2 base	R^2 + TyG	ΔR^2	F	*p*
CV-MAP	0.0216	0.0309	0.0093	17.3	<0.001
MAP-SD	0.0233	0.0346	0.0113	19.5	<0.001
PP-mean	0.0110	0.0122	0.0012	6.7	0.003

B. AUC comparison (binary outcomes)

Outcome	Events	AUC base	AUC + TyG	ΔAUC
CV-MAP ≥P75	1771	0.568	0.582	+0.014
MAP-SD ≥P75	1822	0.572	0.589	+0.018
NE use	597	0.552	0.559	+0.007

Base model: sex + BMI + HTN + DM + duration + ASA + surgery type.

Supplementary Table S7. Sensitivity analyses

Analysis	N	Estimate	Type	95% CI	*p*
Main analysis	7081	0.013	β	(0.010, 0.016)	<0.001
Age <65 only	5223	0.013	β	(0.010, 0.017)	<0.001
Age ≥65 only	1858	0.001	β	(−0.005, 0.007)	0.810
Exclude diabetes	5255	0.012	β	(0.008, 0.015)	<0.001
Exclude hypertension	4729	0.014	β	(0.010, 0.018)	<0.001
Exclude duration >480 min	6975	0.013	β	(0.010, 0.016)	<0.001
CV-MAP ≥P75 (logistic)	7081	1.37	OR	(1.23, 1.53)	<0.001
TyG Q4 vs Q1	3514	0.018	β	(0.013, 0.023)	<0.001

Supplementary Table S8. Age-specific analyses: TyG distribution, post-hoc power, and medication-adjusted results

A. TyG distribution by age group

Group	N	Mean	SD	Min	Max	Q1	Median	Q3
<65	5223	8.991	0.543	6.94	11.05	8.62	8.98	9.36
≥65	1858	9.104	0.529	7.36	11.26	8.76	9.10	9.46

B. Post-hoc power analysis

Group	N	TyG β	SD(TyG)	Power
<65	5223	0.0132	0.543	>0.999
≥65	1858	0.0008	0.529	0.058

Power to detect <65 effect (β = 0.013) in ≥65 group at α = 0.05.

C. Adjusted for beta-blocker and statin use

Group	TyG β	95% CI	*p*
<65	0.0130	(0.0096, 0.0164)	5.5×10^{-14}
≥65	0.0009	(−0.0053, 0.0071)	0.785

Supplementary Table S9. Sensitivity analyses adjusted for antihypertensive and antidiabetic medication use

A. TyG index estimates in medication-adjusted models

Outcome	Model	TyG β	95% CI	*p*	R^2
CV-MAP	Without age	0.0128	(0.0098, 0.0159)	<0.001	0.032
	With age	0.0087	(0.0057, 0.0117)	<0.001	0.073
MAP-SD	Without age	1.076	(0.845, 1.307)	<0.001	0.036
	With age	0.743	(0.514, 0.971)	<0.001	0.081

B. Coefficients for the medication covariates

Outcome	Model	Covariate	β	95% CI	*p*
CV-MAP	Without age	Antihypertensive	0.0072	(0.0026, 0.0117)	0.0020
		Antidiabetic	−0.00003	(−0.0068, 0.0067)	0.99
	With age	Antihypertensive	0.0055	(0.0010, 0.0099)	0.016

		Antidiabetic	0.0006	(−0.0060, 0.0072)	0.86
MAP-SD	Without age	Antihypertensive	0.4906	(0.1456, 0.8355)	0.0050
		Antidiabetic	−0.1391	(−0.6501, 0.3720)	0.59
	With age	Antihypertensive	0.3535	(0.0165, 0.6905)	0.040
		Antidiabetic	−0.0879	(−0.5868, 0.4110)	0.73

All four models included the primary analysis covariate set (sex, BMI, hypertension, diabetes, surgical duration, ASA class, and surgery type) together with antihypertensive and antidiabetic medication use; N = 7081 with no missing data. Age was entered as a continuous covariate in the age-adjusted models (CV-MAP: β = 0.0009 per year, $p < 0.001$; MAP-SD: β = 0.0704 per year, $p < 0.001$). Multicollinearity: the largest generalized VIF raised to the power 1/(2 × df) was 1.90 (diabetes); ordinary VIF values were 1.18 for TyG, 1.95 for antihypertensive use, and 3.43 for antidiabetic use. The antihypertensive coefficient is estimated conditional on a hypertension diagnosis and is compatible with confounding by indication; it is not interpreted causally. The antihypertensive category overlaps with the beta-blocker variable used in Supplementary Table S8. CV-MAP, coefficient of variation of mean arterial pressure; MAP-SD, standard deviation of mean arterial pressure; TyG, triglyceride-glucose; VIF, variance inflation factor.

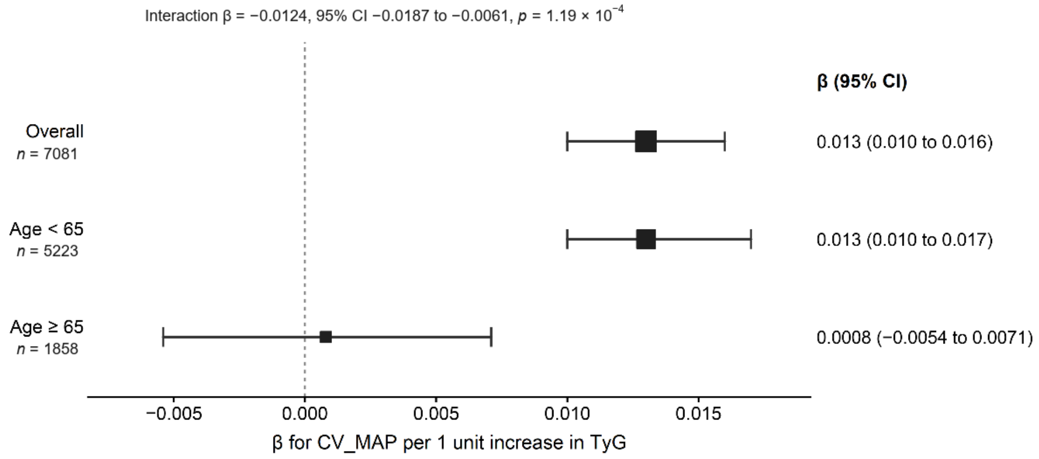

Supplementary Figure S1. Forest plot of the TyG index-CV-MAP association by age subgroup.

Points show the adjusted β coefficient for CV-MAP per 1-unit increase in the TyG index in the overall cohort (n = 7081) and in patients younger than 65 years (n = 5223) and 65 years or older (n = 1858);

horizontal lines are 95% confidence intervals and the vertical dashed line marks the null effect (β = 0). Each estimate was adjusted for sex, BMI, hypertension, diabetes, surgical duration, ASA class, and surgery type. The age-by-TyG interaction was significant (interaction β = −0.0124, 95% CI −0.0187 to −0.0061, $p < 0.001$). CV-MAP, coefficient of variation of mean arterial pressure; TyG, triglyceride-glucose.

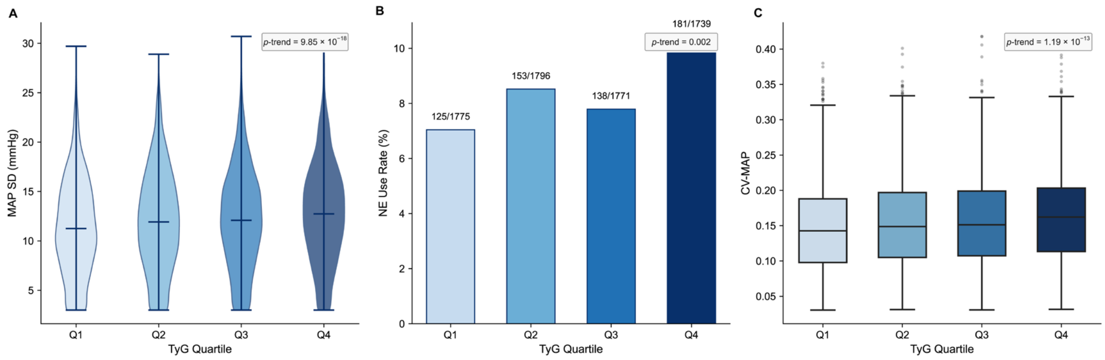

Supplementary Figure S2. Intraoperative hemodynamic profile by TyG index quartile. (A) Violin plot of the standard deviation of intraoperative mean arterial pressure (MAP-SD). *p*-trend < 0.001. **(B)** Bar chart of intraoperative norepinephrine (NE) use rate by quartile. Numbers above each bar indicate the count of NE users and total patients in each group. *p*-trend = 0.0020. **(C)** Box plot of the coefficient of variation of intraoperative mean arterial pressure (CV-MAP). *p*-trend < 0.001. All three hemodynamic indicators increased across ascending TyG quartiles. *p*-trend values were derived from linear regression with quartile rank as an ordinal predictor. CV-MAP, coefficient of variation of mean arterial pressure; MAP-SD, standard deviation of mean arterial pressure; NE, norepinephrine; TyG, triglyceride-glucose.
